# Selections from the Foreign Periodicals

**Published:** 1846-10-01

**Authors:** 


### CIRCUMSPECTIVE REVIEW.

### Selections from the Foreign Periodicals.

New Researches on the Composition of the Blood in Health and Disease. By MM. A. Becquerel and A. Rodier, Docteurs en Medicine.

This paper is the recapitulation of the researches made by these observers during the last year, in the view of confirming, completing, or extending the consequences to which their former labours gave rise to. They have availed themselves of every opportunity which offered itself for analysing the blood in the conditions of the system to be hereafter specified; but never has a venesection been performed with the object of affording them such. The details they enter into as to the various precautions to be taken in conducting the analyses, and the tables exhibiting the results of these, are given at too great length to admit of transcription. We must content ourselves with stating some of the general results, referring those of our readers to the original for more minute particulars.

1. The various descriptions of albuminous matters contained in the blood are endowed with a powerful affinity for water. It results, that when we wish to dry them, these matters yield with great difficulty the last portions of fluid; and, even when they are deprived of these, they instantly commence reabsorbing water from the atmosphere, which is just as difficult of expulsion. The presence of this water, unless the most minute precautions be taken, may interfere in a very material manner with the results of our calculations. Numerous experiments have shewn the authors that to desiccate about 100 grammes of blood or serum continuous exposure for 48 hours to a temp. of about 178° F. is required, and that the powder must be weighed while hot, the instant it is removed from the apparatus, for in a few minutes only water will have been absorbed. The vague expression "albuminous matters" has been designedly adopted, for it is not easy to decide whether it is the albumen or the other materials of the blood which manifest such an avidity for water. Some experiments, however, lead to the opinion that this property especially belongs to the alkaline salts, to free soda, and the extractive matters soluble in water.

2. Blood which has been taken and is exposed to the air is submitted to an incessant evaporation, proportionate to the extent of its surface, the temperature, and the humidity of the air. This concentrates the solid parts, and gives rise to very variable results, only to be avoided by keeping the blood in a vessel hermetically sealed. In the hot days of summer, blood taken in large receptacles, sometimes in this way becomes reduced to at least a third of its volume. It should be received into vessels which are deeper than they are broad.

3. The quantity of serum of the blood determined with proper precautions, is in general proportionate to the quantity of solid matters this fluid holds in solution. The equilibrium may, however, be destroyed. Thus the density is greater when there is little albumen, properly so called, and much extractive matters and free salts. It is less, on the other hand, when there is excess of albumen, and, which is rarer, excess of fatty matters and little extractive matters and free salts.

4. The serum of the blood, whatever its composition, being mixed in different individuals with variable proportions of globules, it follows that, in the complete analyses of the blood, the figures representing the solid matters of the serum have not an absolute value, and we can only compare the relation of the water to these same figures. To obtain an idea of the composition of the serum in health and disease, we must consider it apart, and analyse it separately, after spontaneous coagulation has separated the globules and the fibrine. This important precept was laid down more than 20 years ago by MM. Dumas and Prevost, although other experimenters seem since to have lost sight of it entirely. In all their analyses they have always represented the analysis of the entire blood in one table, and the composition of the serum in another; and it is only in this way we can ascertain the exact modifications of the albumen and other portions in solution.

5. When a bleeding to some extent is practised, and that not too rapidly, the blood has not the same identical composition during its whole progress. The latter portions are more watery and less rich in solid matters. The impoverishment is continuous, although a certain quantity must be lost before it is perceived The diminution of solid parts takes place especially at the expense of the globules, and, to a slight degree, at that of the solid matters of the serum. The proportions of the serum continue much the same. In what manner is this impoverishment to be explained? In the present state of science we believe the explanation of MM. Prevost and Dumas to be the best. "When a small animal," they say, "is bled to a notable extent, the veins absorb with rapidity, at the expence of the remainder of the system, a proportion of liquid perhaps equivalent to that which has been lost, whence it happens that the mass of particles seems diminished in a given quantity of blood." The impoverishment is therefore only apparent, and it is therefore not a matter of indifference at what period of the bleeding we take the blood, the analysis of which is to represent the condition of the system at the time the abstraction is made. It is the first portion that should be chosen.

6. Anterior bleedings exert a sensible effect upon the composition of the serum. It becomes more watery, less dense, and less rich in solid matters. The quantity abstracted and the number of bleedings will much influence this impoverishment, as will also abstinence and the progress of the disease. It is manifested especially in respect to the albumen properly so called, while the amount of extractive and fatty matters and free salts vary little. Pure albumen is the element of the serum which seems to separate with the greatest difficulty. Thus, when a person who has been bled several times becomes convalescent and eats, and the solid parts of the serum are consequently more and more increased, if a new bleeding is practised, as for a complication, the albumen is found to have increased less than any other element. In estimating the effects of prior bleedings we must, however, confine ourselves to short periods, as from 24 to 48 hours, and to the same disease. If a longer period elapses, or the bleeding is employed for a complication, the serum may have repaired its losses, or have become modified by the new affection.

7. We may offer the following statement of the physiological condition of the blood. 1000 of *serum* contain as a mean 90 solid parts. Of these 90 the *albumen* may be represented by 80. The *extractive* matters and free *salts* by 8, and the *fatty* matters by 2. The limits of the physiological condition are 86 and 95: or, much more frequently, 88 and 92. The mean *density* of the serum is 1027, the physiological limits being 1028 and 1026. The highest figures are found in strong persons, in good health, and living well. The influence of age, sex, and temperament is uncertain. In a former essay the authors had given the following as the weights of the principal elements in 1000 parts of *blood.*

Water, 780; globules, 141; fibrine, 22; solid matters of serum, 80. In women the weight of the globules was stated at but 127, the proportion of water being so much the greater. Subsequent researches have confirmed these, every possible care having been taken to select from those persons who applied voluntarily for and insisted upon being bled, only such as gave every sign of health. "Such cases are of rare occurrence, and exact the most rigorous examination, and we have eliminated from them all such as manifested signs of plethora."

8. *Condition of the Serum in various Diseases.* (A.) *Plethora.*—In our former observations we stated that the composition of the blood in plethora did not differ sensibly from that of health, and that the examination of the symptoms rather led us to admit, with some restrictions, the old opinion, that in this condition there is an augmentation of the mass of the blood. Our later researches have entirely had reference to the composition of the *serum*, and have been conducted upon six men and one woman. These prove its composition to be identical with that of health. Its mean density was 1028. The mean amount of solid parts was 91·7, being a little above the physiological mean: but on looking over the various analyses, they are found to be within the physiological limits, although occupying a high position in the scale of these. It is pure albumen, which furnishes the preponderance, the other solids being as in health.

(B). *Simple Fever.*—In four of seven individuals examined the serum did not deviate from the normal state; but in four others its density (1026) was diminished, and the mean of the solid portions was as low as 85—such diminution especially manifesting itself as regards the albumen. This, however, the authors believe rather arises from the abstinence the patients were obliged to observe, than from the effects of the disease.

(C). *Typhoid Fever.*—Analyses have been made of the blood from 25 bleedings performed on 17 persons. In 17 *first bleedings* the density of the serum was low (1026), and it sank in proportion to the gravity of the disease, until it reached 1023. In 7 out of 16 cases the serum was too abundant, in 7 in small quantity, and in 2 normal. In 7 instances it was limpid. The mean of solid matter was only 85·5 to 914·5 water; and the part defective was albumen. In two cases it was normal in amount, and in 1 slightly increased. In 6 *second bleedings* the density was lowered from 1026 to 1024, and the proportion of solid matters to 81. Two patients were bled a *third* time, the diminution still continuing.

(D). *Phlegmasiæ.*—Modern investigation has shewn that, whenever the plegmasia is sufficiently characterized to induce fever, the proportion of *fibrine* is augmented, and is increased, remains stationary, or is diminished, according to the progress of the inflammation.

The present analyses only relate to the serum. Upon 38 patients suffering from well-marked inflammations, venesection was practised 51 times—24 being men, and 14 women. The *first bleedings* produced a diminished density of the blood, especially in severe cases. The density varied from 1030 to 1023. Its mean 1027·5, being of less value in some affections on account of its oscillations. The serum was usually abundant and clear. The mean of the solid matter was 88·4; *i. e.* at the lower limits of the physiological scale; but the amounts varied very considerably. After the *second* bleeding the density was diminished to 1026, and the analyses only gave 81·7 of solid parts. All these effects are increased by repeating the bleeding. In slight inflammations, in those of short duration, and in patients who have taken nourishment up to nearly the time of the attack, the serum may be scarcely at all different to that of the normal state. In severe cases, those which have severely tried the patient's powers, and have required abstinence, the conditions above named are most obvious. Still there are exceptions, but such patients are usually able to take food.

(E). *Pulmonary Tubercles.*—Considered in relation to the alterations which take place in the blood, patients affected with phthisis present different characteristics according to the degree of the development of the organic product. In the earliest stage in some patients the constitution seems not to have in anywise suffered, while in others, an anæmic condition precedes, or is coeval with the tubercles. In the second period, when the tubercles soften, or they are surrounded by inflamed textures, fever is conjoined to the anæmic condition. In the third stage, when hectic, diarrhœa, &c., are present, anæmia, if it has not before appeared, manifests itself. The modifications of the blood, that under these circumstances occur, may be reduced to two principal ones, which may manifest themselves in different degrees and combinations. These are the diminution of *globules* when anæmia is present, and the increase of *fibrine* when phlegmasia complicates the original disease. The condition of the *serum* has been studied by the authors in 16 patients who had undergone 24 bleedings, prescribed for the relief of hæmoptysis, violent febrile action, or some intercurrent phlegmasia. Although, owing to the variability of some of the results, no absolute rule can be laid down, yet, as a general remark, it may be stated that, when any complication existed, anæmia become developed or abundant, hæmoptysis had occurred, the solid portions of the serum diminished in quantity, the water increasing, and the density becoming less—the albumen being the portion of the solid matters in which the diminution occurs. The change of the blood in phthisis bears most analogy to that which occurs in phlegmasiæ.

(F). *Diseases of Spinal Marrow.*—When these are accompanied by paraplegia there is usually a diminution of the quantity of *globules*, and that in proportion to the weakness of the patient and the duration of the disease. In some cases the proportion of globules was less than that observed in some examples of chlorosis, without however any *bruit de souffle* of the carotids being heard. Sometimes the amount of *fibrine* is normal, and at others, if there be an intercurrent inflammation, it may become increased, which occasionally too it does without the existence of any such cause. The *serum* is usually very dense, containing almost always a large proportion of solid matters, as albumen, extrative, &c.

(G). *Bright's Disease.*—Six patients only have offered the means of observation in this affection, and the conclusions drawn from them confirm those published by MM. Andral and Gavarret. These are the small amount of globules: the fibrine normal unless a phlegmasia has supervened: the great diminution of the albumen of the serum (the solid portion of serum varying from 73 to 64), and that in proportion as the disease is chronic in its progress and the bleeding has been repeated. Of course the density of the serum is diminished.

(H). *Pregnancy and the Puerperal State.*—The former researches of the authors were confirmatory of the account of the blood of *pregnant women* furnished by MM. Andral and Gavarret, viz. that the proportion of *globules* is diminished, while that of the *fibrine* is slightly increased. They added another new characteristic, viz. the diminution of the albumen of the *serum,* and the consequently lesser quantity of this fluid. Additional researches confirm these conclusions. The mean density of the serum examined in 13 females was only 1025·8. The mean of its solid portions was but 85. As to the *puerperal states,* M. Andral suggests, in his "Hæmatologie," that the production of fibrine towards the termination of pregnancy is in some manner connected with the phlegmasiæ which so often arise in the puerperal state. The authors in their former essay had referred to two cases, one of eclampsia, followed by puerperal fever, and the other of puerperal fever, in both of which a considerable diminution of the density and of the solid parts of the serum was observed, and they asked whether a diminution of albumen, equal or greater to that in Bright's disease, might not be an essential feature of *puerperal fever.* Since then a M. Hersent has had abundant opportunities of pursuing the investigation, and he has published the result. He states—" 1. That the appreciable changes of the blood in puerperal fever consist in a large increase of water; an extreme diminution of the amount of globules, and an equal deficiency of albumen. 2. That the extent of these changes and the gravity of the disease are proportionate. 3. Probably the vitiation of the blood pre-existed before the explosion of the disease; but that it cannot be considered its cause, although its existence adds very much to the rarity of the case." The authors now refer to several additional analyses they have made in puerperal fevers of different degrees of severity, and conclude with the following observation. "In proportion then as the pregnancy reaches its termination, independently of the diminution of globules which frequently occurs, and the increase of fibrine which is more rare, the albumen of the serum becomes very notably diminished in amount. May we not inquire whether such diminution, which is often excessive, will not explain certain *dropsies* (independently of those induced by the obstruction of the venous circulation by the developed uterus) which appear towards the end of pregnancy; and if the diminution which occurs in eclampsia and puerperal fever may not exert some influence on the production of these diseases?"

(I.) *Chlorosis and Anæmia.*—The condition of the blood has been studied in nine cases of *chlorosis,* in all of which the serum was found superabundant and limpid. Its density was within the normal limits, as was also the proportion of its solid parts, varying from 85 to 89, and furnishing a mean of 87.9. In this disease the changes in the blood had, therefore, reference to the small proportion of globules. In 26 analyses of blood in *symptomatic anæmia* the *globules* were less than 100. The serum was clear, abundant, and of a density of 1026, its solid parts only amounting to 87·7, the loss being in albumen.

The authors thus sum up the results of their examination of the serum in the above-mentioned diseases.

"The density of the serum, and the proportion of the solid parts which it contains, remain within the physiological limits under the following circumstances: viz. plethora, slight or chronic diseases (causing little disturbance of the general system and in which nourishment is still taken), chlorosis, the commencement of pregnancy, and the onset of some acute diseases, &c. In all these cases the proportion, however, approaches the lower limits of the physiological condition.

"The solid portions of the serum, and especially the soluble albumen, are subjected to a very sensible *diminution* under the influence of certain conditions, which however do not at all act in the same manner or with the same intensity. Thus the impoverishment is inconsiderable under the influence of fasting, anterior depletion, and slight plegmasiæ. It is more marked in severe disease, especially when this is prolonged, in severe phlegmasiæ, typhoid fever, sympathetic anæmia, the termination of chronic disease, the end of pregnancy, &c. Lastly, it is extremely great in Bright's disease, puerperal fever, and certain diseases of the heart producing dropsy.

"The *increase* of the proportion of solid matters of the serum is a very rare event. It is found in too isolated cases to admit of any general rule being laid down. It is, however, nearly constantly observed in diseases of the spinal-marrow."—*Gazette Medicale,* Nos. 26, 27, 32 and 36.

---

### The Cæsarian Operation considered in a Theological point of View.

Some months since a woman died in Brittany, being about six months advanced in pregnancy. The parish priest sent for a medical man to remove the child by the Cæsarian section, in order that it might be baptised, if still alive. Upon his refusing to do so, the priest sent for a *farrier,* who removed a dead fœtus by the Cæsarian section. These facts being severely commented upon in some of the newspapers, the minister published a reply in justification of his proceedings, stating that they were imperative upon him, in proof of which he quoted Bishop Bouvier's *Sacred Embryology*, which is a text-book of the church. This expressly orders the section to be made as speedily as possible, under all circumstances as regards the age, time, and mode of death, &c. and in respect to the person who is to be called upon, it says—" No endeavour must be neglected to procure the services of a professional man. If this is impossible, a midwife, some other woman, a married man, or in a case of urgency any one at hand must be resorted to, but *never a priest*, unless there is absolutely no other person who can be procured." When the medical man refused, the priest read these and other passages of M. Bouvier's work to the farrier, shewing him the absolute necessity of proceeding with the operation.

A medical man, before resorting to the operation has to satisfy himself—1, That the *woman is really dead;* 2, that the *child is alive*, and 3, that it is *viable*. This restricts obviously the operation within very narrow limits. The Church regards not the *viability* of the child, but the chance of its being alive, and therefore fitted for baptism. But the chances of error and danger that this rule gives rise to are obvious. Is the practitioner to submit to the dictates of the priest and open the woman's body the instant she has yielded her last breath? Many there are who would hesitate to do so, and if the professional man delays in order to be certain that his patient is really dead, does it seem reasonable to allow persons utterly destitute of physiological knowledge to take upon themselves not only to judge of the propriety of the operation, but even its execution? This has been so strongly felt by some of the clergy themselves even, that various contrivances have been thought of to dispense with the necessity of the operation, and yet confer the rite of baptism upon the infant. Thus, *vagino-uterine injections* have been advised, and, quite recently, these have given rise to a learned discussion in the Academy of Medicine of Belgium. The validity of such a procedure has been admitted by high authorities of the Church, so that, even according to its own showing, the necessity for the operation may be brought within very narrow limits. When, however, it is determined to practise the operation this should be solely confided to professional men; for an apparent state of death during gestation is of too frequent occurrence, and of too difficult detection, to allow of the interference of unskilful persons. When medical men are regularly consulted in these cases, they should bear in mind the duties and obligations of their profession, and lend their assistance, when the rights of humanity, of which they are the natural guardians, do not oppose, to the accomplishment of the duties of religion, in the same manner as they are accustomed to do with regard to those of justice.—*Gazette Medicale*, No. 30.

[We cannot trust ourselves to remark upon this example of priestly interference; but we ask, is the period of its occurrence the nineteenth century, and the country, enlightened France?—*Rev.*]

---

### On the Treatment of Photophobia. By Dr. Duval.

In those ophthalmias which are attended by an exaltation of the vital properties of the organ, we first combine antiphlogistics with the remedies which are calculated to reduce preternatural sensibility. When the inflammatory action has subsided, we continue this latter class of *antineuralgic* remedies alone, varying them from time to time, as, by the persistence in the use of the same one, its action becomes enfeebled. Another important indication in their use is always to proportion their dose to the severity of the affection. A too energetic antispasmodic, it is true, throws the part for a time into a state of torpor, but re-action comes, and we only induce a more severe exacerbation.

*Opium.*—This is one of the most useful remedies in affections of the eye when photophobia is present, the pain coming on in the paroxysmal form. From 3 to 6 grains should be dissolved in water and rubbed into the supra-orbitar region three or four times in the twenty-four hours. Mackenzie especially recommends a combination of calomel and opium given internally in cases of photophobia arising from rheumatic and catarrho-rheumatic ophthalmia.

*Belladonna.*—An infusion of the dried leaves given internally is a far more certain and efficacious remedy than the extract, &c. is used externally. Ten grains of the infused leaves repeated every three or four hours will often produce great and speedy ease: but the increase of this quantity must only be very carefully decided upon, as it will, if carried too far, produce symptoms of poisoning. The earliest symptoms indicating the necessity of suspending it are a desire to spit, a dryness of the mouth, inspissation of the saliva, and a burning sensation in the throat. After this, the brain becomes confused, giving rise to hallucinations and afterwards to cerebral torpor. Some practitioners give a mixture of *extract of belladonna* (one or two grains per diem) and *sulph. quinine,* either by the mouth or in a lavement. M. Rognetta speaks highly of its combination with *calomel* for the photophobia of scrofulous ophthalmia. *Ext. Bellad.* gr. $\frac{3}{4}$. *Calomel,* gr. xv. Divide into 12 pills and take one three times for the first day, and two for each dose next day, and so on. In this way the belladonna has been sometimes increased to 4 gr. per diem with impunity, although the cause for its use usually disappears after the first or second day. When using the extract belladonna *externally* M. Duval mixes it with a double quantity of *mercurial ointment,* adding, if the pain is very obstinate or acute, from three to ten grains of opium. If the ointment is to be continued the proportion of belladonna must be augmented. About a scruple should be rubbed into the temples and forehead four times a day. He prefers the watery to the alcoholic extract, the latter being liable to fermentation, during which its anti-neuralgic properties are dissipated; and indeed, united with alcohol, the belladonna seems to acquire even a stimulant property, at all events for a while after its application. The extract of the green leaves is more active than that made from the dry ones: and that procured by displacement is to be preferred.

*Hyoscyamus* is usually considered succedaneous to belladonna, but its utility is doubtful except in very slight cases, and its dose should be double that of the latter. *Stramonium* is more energetic, and must be given in half this quantity. "To both we prefer Belladonna, which we employ, whatever be the extent and nature of the ocular inflammation, providing photophobia be present."

*Camphor* is of great service in essential photophobia the consequence of very prolonged ophthalmias (opthalgia), or coming on after a prolonged sojourn in a dark place. It should be dissolved in alcohol or mixed with mercurial ointment, and applied over and near the region of the eyebrow and temple. Inspiration of camphor is also highly useful.

*Excision of the Conjunctiva.*—When the above measures fail in giving relief, and especially when the membrane is vascular or villous, we have recourse to this operation. The relief by this little operation is so great that we may term it an antiphotobic measure, par excellence, and recommend it, whatever part of the eye may be affected, or whatever the nature or degree of the ophthalmia. It causes little pain and is easy of execution. We employ Pellier's elevator, Maunoir's forceps, and scissors having a very slight curvature. Seizing a fold of the conjunctiva near the conjunction of the cornea and sclerotica, we make a circular excision of this membrane. This little operation, usually reserved for chemosis only, has always succeeded in our hands in those cases of obstinate photophobia dependent upon chronic ophthalmia—but it may be resorted to with equal advantage when the photophobia is excessive in acute ophthalmia.

*Puncture of the Cornea.*—M. D. has performed this operation seven times with most remarkable relief to the patient's sufferings. They were all examples of various forms of ophthalmia, and all tormented with the intensest photophobia. After treatment, appropriate to their cases, had been adopted, they all resumed their usual occupations. He believes this operation has been too much neglected, nor has he found the inconveniences in its performance indicated by Gregory.—*Annales d'Oculistique,* Tom. XI. p. 101-111.

---

### Cause of Death in the Aged.

According to M. Rostan the most frequent cause beyond all others is the obstruction of the circulation induced by arterial or valvular ossifications. He has had abundant opportunities for pursuing the investigation at the Salpètriere, and has found that ossification of the sigmoid valves of the aorta, and frequently of the auriculo-ventricular valves, as also of the arterial walls themselves, existed in almost all the subjects of advanced age, whose physiological condition it may in some sense be said to be. Hence the obstruction of the circulation, the consecutive congestion of the viscera, and the necessary death. Boerhave stated that senile deaths occurred through affections of the brain, and Bichat, that they might arise from interruption of the circulation, the respiration, or of the action of the brain. M. Rostan, however, believes the natural, senile, so to speak, physiological, death always occurs in consequence of the changes in the heart and arteries mentioned. Some authors believe ossification to be the result of inflammation, but that this is not always the case, may be seen from the fact of its frequent occurrence in those aged persons.—*Gazette des Hôpitaux.*

---

### Velpeau on Fracture of the Olecranon and Patella.

" Some of the observations we find delivered by our classical authors on these accidents are true only in exceptive instances. Thus, they tell us, inevitably a great separation, amounting to an inch or more, takes place, while in fact the interspace does not usually exceed a few lines. The prognosis of these cases is not serious ; for even those we meet with which have not been treated at all, do very well. In the case of the olecranon there is only an impossibility of making complete extension, and of the patella some weakness of the limb—the fibrous matter which is deposited being in fact almost as solid as bone itself. If we had no other means to recommend than that of two months' immoveability of the limb, we had better not attempt treatment at all, for more will be suffered from the consequent stiffness and œdema than from the injury itself. The limb should, however, be placed in moderate extension, the figure of 8 bandage applied, and over it the dextrine bandage for three weeks or a month.

" To shew that I do not exaggerate the harmlessness of these accidents, I may observe, that very considerable movements of the part are retained, even when the separation of the fragments is considerable. Thus, I have seen a Belgian diplomatist who was able to walk about, although the patella was drawn up to almost the middle of the thigh. A ship-captain, the fragments of whose patella were separated by four or five fingers' breadth, scarcely limped. Nor are the consequences more serious in fracture of the olecranon."—*Gazette des Hôpitaux.*

---

### On the Law of the Formation of Primary Abscesses external to the Bone (as distinguished from consecutive sub-periosteal abscesses, dependent upon suppurative inflammation of the medullary canal) after Compound Fractures (by Contrecoup) and Dislocations of the Long Bones. By Dr. Laugier, Surgeon to the Beaujon Hospital.

Attention has been sufficiently directed to the injury which the muscles, capsule, blood-vessels, &c. undergo in these accidents, while the effects of violence upon the soft parts immediately in contact with the bone, but *opposite* to where its displacement has taken place, have been overlooked; and yet I do not hesitate to say that in the production of the primary or consecutive, and frequently fatal, mischief these lesions play a most important part, although they may exist for some time undetected. The truth of this opinion is proved by the surprising constancy with which abscesses consequent on these injuries are found so situated. The reason of it is that the laceration and separation of the soft parts from the bone are far greater upon the opposite side to that on which the displacement occurs, although the fragment of the bone may penetrate the muscles and skin on this last. If the reduction be not complete, a space between the bone and soft parts persists and becomes the centre of tedious suppuration, the discharge, which had it occurred on the side of the wound would have easily escaped, now burrowing along the course of the bone or bones, and inducing a yet greater separation of the soft parts before any external manifestation of the existence of the abscess is afforded. It is remarkable to what an extent published cases of compound fracture are defective in details concerning the seat of abscess sufficient to test the accuracy of this statement—the observer usually contenting himself with stating the fact of abscesses having existed without indicating their locality. Where such statements have been furnished they do not however impugn its correctness. The same law applies to compound dislocations; for it is at the side opposite to that on which the head of the bone has been displaced that the principal seat of inflammation and suppuration is found.

Several years since I demonstrated that, as regards dislocations of the first metatarsal bone, when abscess follows, it always forms on the outer side of the reduced metatarsal, and that, to prevent it, it is necessary to make a deep incision parallel to the external side of this bone. I soon discovered that this was but an example of a general law equally applicable to other fractures and luxations of the long bones: and the knowledge of such a law has enabled me to preserve many limbs which would otherwise have been amputated, or in which the consecutive lesion would have proved mortal. Guided by it I have made a *preventive incision* at the appropriate spot, or directed the various local applications, as leeches and fomentations, and at a later period, suitable pressure, to be made there.

Some persons have seemed to think that the above views were already comprehended in the rules laid down for making preventive counter-openings at the base of a flap in certain wounds of the head. But my remark does not relate so much to the propriety of making preventive incisions as to the indication of their proper locality; and if the analogy above alluded to really exists, at all events it has never before been perceived; and in fact no author has indicated hitherto the seat of election of deep-seated abscesses after compound fractures and dislocations of the long bones. The principal, and indeed the only, point I have in view to prove, is, that these *primary abscesses are situated on the side opposite to the displacement.* As to consecutive abscesses occupying another seat, they are only found when the recognition of the primary one has been neglected. The pus burrowing around the bone spreads out in whatever direction the aponeuroses allow it a free course. Such an abscess observed at a more or less considerable period after the accident, would therefore in no-wise invalidate the law now stated, and so much the less so, inasmuch as these secondary abscesses communicate with the primary one, whence they have derived their existence.

But do *simple* fractures by *contre-coup* and dislocations offer nothing analogous? They do; only from the much less separation and injury of the soft parts in these accidents, the occurrence of abscess is much less common: but when it does happen, like the chief injury to the soft parts, (which indeed in simple dislocation is often very considerable, giving rise to relapse, paralysis, &c.) whence it arises, it is also situated at the external part of the limb.

Dr. Laugier briefly relates the particulars of a few cases occurring in his own practice exemplifying the law he is endeavouring to establish; and quotes two or three from the remarkably small number that have been recorded in the periodicals in sufficient detail.—*Archives Generales,* tom. xi. p. 133-145.

[Although we think "preventive incisions" must not be too hastily resorted to for averting evils which may perhaps never occur; yet, if the above-stated circumstance proves upon further examination to be indeed a law, it will induce more careful examination of the suspected locality than is now usually made. —*Rev.*]

---

### Memoir on the Paralysis of the Third Pair of Nerves consecutive to Neuralgia of the Fifth Pair. By M. Marchal (de Calvi).

My object is to signalize a relationship which exists between paralysis of the third pair with neuralgia of the fifth that has not been hitherto suspected. Trifacial neuralgia has been little studied as regards the disorders which it produces beyond the nerve it affects, but which form a very interesting and curious part of its history. Is it not in fact remarkable that a lesion, limited to a few filaments of the fifth, can, by a retrograde repetition of morbid actions, propagate itself to the nervous centres and induce the most extensive, multiplied, and serious accidents, such as the loss of speech or power of deglutition, excessive dyspnœa, paraplegia, violent convulsions, emprosthotonos, furious delirium? Nevertheless this is to be found detailed in a case by Ponteau, which I have published with several others in a paper upon *Traumatic Prosopalgia* in the 55th Vol. of the *Memoirs of Military Medicine.* And in these so certainly was it the simple lesion of some of the trifacial filaments that induced so fearful an assemblage of symptoms, that when they were divided, by a section extending to the bone, the accidents, which had so long resisted all medical appliances, disappeared in half an hour never to return. Two phenomena or two orders of phenomena are sometimes so disproportioned that the idea of their connexion never at first presents itself to the mind; for who could have thought such grave disturbances of sensibility and motion were dependent upon an old contusion of a few nervous filaments. Several facts and a careful examination of all their circumstances were required before this connexion could be perceived. These cases of prosopalgia, with *general* lesion of sensibility and motility, led me to recognize the *special* relation which exists between the paralysis of the common oculo-motor nerve and neuralgia of the trifacial, in the following cases.

Case 1.—A soldier æt. 47, of a very nervous temperament, was the subject of paroxysmal pains of dreadful violence on the left side of the head and face, especially in the vicinity of the supra-orbital foramen, mastoid process, and in the teeth of the upper jaw. The left eye became affected also with diplopia, but presented no deviation from its normal direction. The sensibility of the left cheek was entirely gone, as also of the nostril, although he could still perceive odours. He could open his jaws only to a very slight extent. M. Marchal tried the experiment of compressing the frontal nerve as it passed out of its foramen. This caused great pain, *but immediately, and as long as it was continued, the diplopia ceased.* The experiment was frequently repeated with the same results. The pressure, however, could not be employed as a remedial means in consequence of the great pain it gave rise to: but the patient obtained considerable ease during his paroxysms from inducing compression of the dental nerves by introducing a small piece of wood between two of his teeth. Seven blisters were successively applied over the supra-orbital region in the space of 20 days, purgatives and stimulating pediluvia being simultaneously resorted to. The pain was relieved and the sensibility restored; but the diplopia remained and the globe of the eye became smaller, and drawn inwards, the upper eyelid being also paralysed, so that the eye was kept shut. But now analogous pains and diplopia were observed on the right side—so that this latter could no longer, as heretofore, be obviated by closing one eye. Blisters were applied on this side and the pain relieved: but the diplopia of either eye continued, and the patient's vision became sensibly enfeebled. Time and the use of Meglin's pills, or probably the first alone, gradually restored his vision; and, one evening, after drinking to excess, the diplopia also suddenly left him. The patient, however, eventually became the subject of various other nervous affections, which entirely destroyed his health.

CASE 2.—A young woman, æt. 26, and otherwise in perfect health, had suffered for two years most violent pains in the left side of the head, radiating towards the ear, eye and cheek. They were accompanied by tinnitus auris and red flashes before the eye. Eight days before visiting her the eyelid could not be raised, and the globe of the eye was simultaneously drawn outwards. The pupil was dilated. A sharp pain was felt opposite the supra-orbitar foramen, and increased when she laid on that side. Following her occupation as a shoe-binder, she had many years since been accustomed to press the left side of her head against an article of furniture. This gave rise to a tumour here which suppurated, and the resulting sore was obstinate in healing. On touching the cicatrix which this left a sudden and violent frontal pain was felt. Blisters were applied over the cicatrix, and galvanism employed in the course of the third pair, but all without success.

CASE 3.—M. Marchal having accidentally observed a pensioned soldier with a blephoroptosis, inquired its history. He learned that the man, after having been exposed to damp, had suffered horrible paroxysmal pains at the root of the nose and near the supra-orbital foramen. After a certain time these ceased, and were followed by the complete descent of the eyelid, the globe of the eye being also drawn outwards and the pupil dilated.

CASE 4.—Louise Heberard, æt. 33, had enjoyed good health until she worked as a dress-maker in a cold, damp, apartment. In June, 1844, she was seized with tooth-ache on the left side, and then with pains along the left eyebrow, and eventually opposite the supra-orbitar foramen. Severe pains were also felt at the root of the nose, and near the angle of the jaw. The left eye became drawn inwards and she saw double. In May, 1845, the upper eyelid fell, and the eye which had been drawn inwards now became drawn outwards. Tactile sensibility of the left side of the face and head was abolished. The sense of smell was gone on the left side, as also that of taste at the anterior part of the tongue. During mastication, the patient often bit the left side of her tongue, and she articulated so imperfectly as to be understood with difficulty. She was much troubled with confusion of the head, and could not guide herself unless the left eye was closed, no account of her double and confused vision. No means that were tried gave her more than partial relief and she left the hospital.

Case 5.—A man in M. Gendrin's ward, while employed on a railway, had received a blow upon the forehead, which induced violent pains radiating towards the surrounding parts. Upon his admission, long after the accident, pressure upon this point still caused some pain; and, several months after the existence of these neuralgic pains, the upper eyelid of the same side fell, and the eye was drawn outwardly.

"In these cases it cannot be doubted that the neuralgia of the 5th pair preceded the paralysis of the 3rd. As in the third case, the neuralgia may have ceased for a longer or shorter space of time, and then the paralysis may seem to be independent of it, until due enquiry is made. I am certain that a great number of cases of paralysis consecutive to neuralgia may, in this way, be detected."

M. Marchal believes the following hypothesis offers the most probable explanation of the occurrence. The trifacial nerve and the common motor oculi meet in the ophthalmic ganglion, the former furnishing it the sensitive root by the nasal branch, the latter the motory root from its inferior branch. "It will be admitted that a reflex morbid action may take place within this ganglion by which the affection which is expressed in the sensitive nerve by pain or anæsthesia, is transmitted to the motor nerve, in which it is expressed by convulsion or paralysis. I say convulsion: for, in the first case, the eye was drawn inwards as it also was at first in the fourth. The symptomatology of the motor, as of the sensitive nerves, is of two opposite kinds; pain and anæsthesia for the latter, convulsion and paralysis for the former; and in this way, prior to the paralysis of the rectus internus, it may have been in a state of excitement, during which the eye would be drawn inwards."

This hypothesis is consistent also with the most plausible theory of the functions of the nervous ganglions—true miniature brains, as they have been called, for the regulation of special actions—receiving impressions by filaments continued from the sensitive roots, and conveying these by the motory filaments—presiding over the nutritive phenomena by their grey fibres, and only advertizing the brain proper of what is occurring in their localities, under extraordinary circumstances. In this way, the ophthalmic ganglion, in particular, would be affected in the relations prevailing between the retina and the iris, and certain muscles of the eye. Advertized of the vicissitudes of sensibility of the retina by its connection with the optic nerve, it reacts upon the iris, harmonizing the pupil according to the degree of sensibility of the retina, and acts reflectively by its motory root upon the muscles of the eye which are influenced by the third pair.

There is then, besides the perception belonging to the brain, another, viz. a *ganglionic* or *organic perception.—Archives Generales,* Tom. xi. pp. 261-273.

We think it may prove advantageous to place in juxta-position with the foregoing interesting paper an abstract of the following

### Case of Amaurosis of the Right Eye from a slight Wound of the corresponding Eye-brow. Related by Drs. Michelacci and Fedi.

The Reporters observe that it is still a matter of controversy whether a simple traumatic lesion of a branch of the fifth pair can induce amaurosis. Muller seems disposed to attribute its production to commotion of the retina or the optic nerve, although there certainly exist examples of amaurosis following severe lesions of the forehead, without any such concussion having taken place. Malgaigne, too, trusting to a false maxim that the lesion of a nerve may paralyse its terminal branches, but cannot operate in a reverse manner towards the trunk, is likewise intent upon proving the ease with which the peculiarity of the structure of the orbit allows of the production of commotion of the optic nerve. Lawrence doubts whether amaurosis ever results from injury of the frontal nerve.

The example we here adduce is not explicable, at all events, upon the above supposition. The patient became amaurotic immediately after receiving a small wound from a shot over the right eyebrow. Three questions were proposed by the legal authorities for the consideration of the Reporters. 1. Whether blindness really existed? 2. Can it be referred to the infliction of this small wound? 3. What hope is there of a cure? For a reply to the first of these the state of the patient's eyes was diligently examined: and they were found to be quite natural in appearance, as also in the action of their pupils as long as both eyes were kept open: but when the left eye was closed the right pupil was found to be quite unobedient to any stimulus whatever. "The results of the experiments we tried proved, in our opinion, the perfect blindness of the right eye, depending upon a complete paralysis of the sensorial nerve. The movements of the iris of the blind eye, which took place whenever the light was allowed to exercise its influence upon both eyes together, or only on the left one, do not at all depend upon the sensitiveness of the right retina, but are exerted solely by virtue of the nervous action excited by the light in the left eye, and by its sensorial nerve reflected through the medium of the brain upon the motor nerves of the right iris. These results agree with those I have observed in other cases of amaurosis confined to a single eye, and find their explanation in the doctrine of the reflex nervous action, as taught by those celebrated men Marshall Hall and Müller."

A very small cicatrix was observed over the orbital ridge, just at the point where the frontal nerve emerges from its foramen; and the blindness having immediately supervened upon the infliction of the wound which produced this, the second question was answered in the affirmative.

The prognosis was unfavourable; for seeing the rapidity with which the blindness was induced, the completely amaurotic condition of the visual apparatus, and the long period which had elapsed (34 days) without any improvement having resulted, and recollecting Scarpa's opinion upon the rarity of cure in these cases, it is to be feared the loss of the sight of the eye will prove permanent.—*Annali Universali,* Vol. 116, pp. 21-32.

[Agreeing with the Reporters that cases enough are on record to allow of the admission of the production of amaurosis by injury to the frontal nerve without concomitant *concussion of the retina,* we cannot allow that their own case, one of gun-shot wound of the forehead, although a slight one, can be considered as an unexceptionable example of this.—*Rev.*]

---

### Observations upon Electrical or Acute Chorea. By Dr. Dubini.

During the last ten years Dr. Dubini has observed about thirty cases of this dangerous form of chorea, and his colleagues have met with a great number also. He seems to have imposed the name "electrical" upon it in consequence of the resemblance which the muscular movements bear to those produced by the electrical shock. One finger, one extremity, or one side of the face (generally the right) only is first affected, but in the course of a few days the whole of the *same* side of the body becomes implicated. The movements are almost constant, and, besides those of a more moderate character, there occur, two, three, or more times in the twenty-four hours, violent convulsive paroxysms affecting precisely the *same muscles,* accompanied by a rapid pulse and followed by profuse sweating.

After these have subsided the parts affected by them fall into a more or less complete state of temporary paralysis.

Fear seems in the majority of cases to have induced the disease, although, in some, the patients who are usually young (from 7 to 21) and robust are unable to trace it to any such cause. In some instances, pain in the head or in the course of the spine have preceded the attack. The convulsive action always attacks during the course of the disease precisely the same identical muscles, producing oftentimes great distortion of the eye, mouth, head, &c. Occasionally certain fingers, or one hand only may be affected without the disease extending to the rest of the body for 30 or 40 days, and in one case the only symptom consisted in an affection of the muscles of the tongue for nine days; but the slightness of the attack, and the otherwise apparent health of the patient, must not put us off our guard, as the case is just as dangerous as the more severe forms of the disease. During the convulsive paroxysms the patient articulates with great difficulty, and if he has not arrived at the stage of stupor, he is usually employed in steadying the one arm, which is agitated just as if by electricity, with the other. The limbs are usually tumid but not œdematous; and the surface is morbidly sensitive, so that the least touch of the arm will sometimes excite the most violent clonic spasms of the whole corresponding side. As the case gets worse the convulsions become more unintermitting and sometimes implicate the other half of the body. They, however, get feebler and at last cease. The patient now becomes comatose, sweats pour from the body, the eyes sink in the head, the respiration is stertorous, the pupils are fixed, and the cornea becomes softened and sometimes ulcerated from the pressure at the angle of the eye. The pulse, which during the existence of the convulsions had been strong and vibrating, is now feeble or imperceptible. This condition may precede death for a day or two. The intellectual faculties are preserved, as indicated by the movements of the patient's head, when the convulsions render speech impossible, until the apoplectic stage of the disease; and even in young children a remarkable presentiment of the fatal issue of their disease is often observed. The appetite is at first good, but becomes gradually less. The bowels get confined, and almost all the patients void *lumbrici*. Those of them who do not pass the worms, however, have not the disease milder than the rest—nor does the expulsion of these by anthelmintics give any relief.

The disease may continue from one to five or more months, if antiphlogistic treatment, suggested by the early deceptive and transitory symptoms, does not hasten the fatal termination.

Diligent examination of the head and spine after death only exhibits slight venous meningeal congestion, the quantity of limpid serum not being anormally large. No lesion of the cerebral substance is observed, save in a few instances some bloody points upon slicing it. A much greater degree of venous congestion is usually found, when bleeding had not been practised, in the chest and abdomen. Where bleeding has been resorted to, the brain has been found in a state of complete anæmia. Dr. Dubini found in three cases (out of 30) softening of the thalami optici.

The author regrets that, as regards remedial measures, he is obliged to confine himself to stating what he has found useless—the disease usually proving intractable; and the only two cases which did recover, did so in the employment of measures which in other cases had proved useless. As necroscopies do not, however, exhibit organic changes as an essential part of the disease, he hopes that one day a successful mode of treatment may be devised. *Antiphlogistics* are contra-indicated, and even the depression produced by a purgative or by the menstrual flux has aggravated the symptoms. Anthelmintics, large doses of Calomel, Oxide and Valerianate of Zinc, Opium, Strychnia, Quinine, Iron, and Belladonna have been in turn tried in various cases without satisfactory results. The best palliative of the violence of the convulsions seems to be *Extract of*

*Hyoscyamus:* and Dr. Rotondi reports three cases to the author in which a definite cure even seems to have resulted from the use of large doses of this substance.

Dr. Dubini enters at great length into the consideration of the differential diagnosis of this disease. He observes that violent muscular contractions characterize many diseases, but he has not been able to find the assemblage of symptoms just described in any work he has consulted. They may be thus recapitulated. Fear the common cause of the disease—the movements like those resulting from electricity—the same identical muscles affected, and all those situated on the same side of the body becoming gradually implicated—the same muscles after undergoing these violent contractions fall into a state of temporary paralysis—apoplectic stupor supervening upon the frequent repetition of the convulsive paroxysms—the absence of lesions explicable of the symptoms, and the fatal termination. He presents his readers with detailed synoptical tables of the differential diagnosis of this disease and ordinary chorea, epilepsy, eclampsia, ergotism, saturnine convulsions, cerebro-spinal meningitis, and convulsions from organic disease of the brain. We have only space for the transcription of the first of these.

| *Chorea Gesticulatoria, or St. Vitus' Dance.* | *Chorea Electrica.* |
|---|---|
| 1. The disease is characterized by irregular partial or general movements, changing at every instant, and giving rise to involuntary and frequently ridiculous gestures. Of the two sides of the body the left is attacked in preference: so that, of 25 cases observed by Rufz, in only one were the extremities of the right side affected. The irregularity of the muscular contractions is the pathognomic sign. | 1. The movements, strikingly resembling those resulting from electrical shocks, are always identical, constantly affecting the same muscles as at the commencement, gradually however implicating others of the same side also. Usually one side only affected, and that the right one. The identity of the muscular contractions is the pathognomic symptom. |
| 2. The patient, at first of an active and correct frame of mind, may become fatuitous, or acquire a marked mobility of character, by reason of which he laughs or cries, &c. without apparent cause, or falls into a state of apathetic indifference, the result of debility of intellect. | 2. The patient seems aware of the gravity of the disease, and has for the most part an invincible presentiment of its fatal termination. No change of character is observed. |
| 3. The vague movements of chorea are not necessarily followed by paralysis. If the patient sometimes cannot govern his movements in walking, that is rather due to the irregularity of the muscular movements than to the existence of paralysis. | 3. The muscular contractions are followed insensibly, and sometimes suddenly after the first paroxysm, by a paralysis of the affected parts. |
| 4. The disease in the majority of cases terminates in a spontaneous cure, or it may be relieved by various remedies. If death does occur, it is always in consequence of an inter-current disease. | 4. Almost all the cases terminate in death, in spite of the most varied means of treatment—so that it may be literally said that death is the rule, recovery the exception. Death, moreover, is due to the progress of the disease, which, by becoming more and more general, induces apoplectic stupor and death. |

*Annali Universali,* Vol. 117, pp. 1—32.

### Treatment of Fracture of the Clavicle.

Two difficulties are met with in the treatment of this accident; the maintenance of the fragments of the bone immoveable, and the procuring a regular callus. Desault's bandage does not entirely fulfil these conditions, but M. Blandin obtains them by rendering this apparatus immoveable by means of dextrine. Six cases have just been treated by him at the Hôtel Dieu, and in one only, this patient being delirious from a wound of his head, was the success incomplete. In the others, when the bandage was removed, it was difficult to point out the side upon which the fracture had occurred, no angle or projection whatever being visible. It might be supposed that an immoveable bandage of this kind would prove very irksome to the patient. It does not: for, owing to the fragments being maintained *in situ,* he suffers less; and there is no necessity to renew or tighten the apparatus every few days, as would otherwise be the case. Care is of course taken to guard the armpits, and other parts which the stiff bandage would irritate, by means of compresses. In private practice, M. Blandin causes the patient to wear a somewhat tight flannel-waistcoat, over which he applies the bandages; and with the skin thus protected, the most delicate females experience no inconvenience from it.—*Gazette Medicale,* No. 33.

---

### Nitrate of Silver in Traumatic Erysipelas, Arthritis, &c.

Erysipelas frequently shows itself in the St. Louis Hospital, and it is always treated locally (employing purgatives, &c. according to the indication) by M. Jobert with the *nitrate of silver ointment,* of varying strength, (viz. 4, 8, or 12 parts of the nitrate to 32 of the lard,) according to the degree of action desired to be obtained. He also uses this very advantageously in acute and chronic inflammations of the joints, bones, periosteum, and cellular tissue; violent arthrites, white-swellings, &c. The inunction quickly dissipates the inflammatory symptoms, and especially the pain, so as to be regarded by M. Jobert as one of the most powerful antiphlogistics. The application of the ointment blackens the skin. This becomes less stretched, wrinkles, and undergoes a kind of desquamation. The blackened epidermis is gradually detached, and the skin resumes its normal colour. When the inflammatory symptoms have disappeared, the disappearance of the blackening may be hastened by washing the skin with a solution of ioduret of potassium. In persons possessing a very delicate skin slight pustulation, or a pungent pain of short duration, may be produced.—*Gazette des Hôpitaux,* No. 96.

In connexion with the above subject, we may mention that M. Robert employs *sulphuric acid* with great advantage in *chronic arthritis.* He dips a small pencil in the acid, and makes four or five linear superficial cauterizations. Such marked relief from pain results, that the patients themselves call for a repetition.

M. Hilsenberg has likewise employed the same means after local depletion with equal advantage. He however uses the following formula: *Acid. Sulph. Concent.* 8 *parts, Syrup* 15 *parts.* To be rubbed in twice a day.—*Abeilie Medicale.*

---

### M. Velpeau on Flexions and Engorgement of the Uterus.

"A proof of how often the term *engorgement* has expressed an error of diagnosis is found in the fact that of late years, and in proportion to the progress of science, engorgements of the uterus become more and more rare, while the number of vicious flexions is augmented. I do not fear to state, that of 50 women reputed to have uterine engorgement, 45 will be found upon examination to be suffering from some deviation from the normal position of the organ. How can we explain this error of diagnosis being committed by well-educated practitioners? The reason is simple. The woman is examined in the recumbent posture, and the finger meets, in a certain direction of the neck, forwards if there be anteflexion and backward if there be retroflexion, with a tumour of considerable size and sensible, which is declared to result from an engorgement. But the tumour is simply the body of the organ bent at a more or less obtuse angle, and sometimes at a right angle. We can easily assure ourselves of this, especially when, as is the case with most women who have borne children, the walls of the abdomen are neither tense or thick. By gently depressing the hypogastrium, we may grasp the body of the womb between the two hands, and appreciate its volume as accurately as if we could see it. Engorgement is one of the least usual conditions of the organ that we meet with, and on the contrary the body of the organ is often found somewhat atrophied.

"Accurate diagnosis, in consequence of the treatment it designates, is here of great importance; for when we have to do with a simple deviation we dispense with the use of means proper for resolving a tumefaction which does not exist, of debilitating remedies each more mischievous than the other, and with confining the patient for months in the recumbent posture. We order for her, moderate exercise, a substantial and tonic regimen, antispasmodics, ferruginous drinks, saline baths, astringent vaginal injections, and lastly an abdominal bandage which may support the viscera and prevent their weighing down the deviated organ."—*Gazette des Hôpitaux,* No. 89.

---

### On Paralysis of the Insane. By M. Baillarger.

Incomplete general paralysis is an apyretic disease, generally of long duration, and principally characterized by embarrassment of speech, the *progressive* paralysis of the limbs, and dementia. Its exact frequency cannot be determined, as many patients who are the subjects of it are retained at home by their families; while it is also often overlooked by medical men, or confounded with diseases of the brain. About one-sixth of the patients admitted into the large asylums of France are affected with this paralysis. More than one-half the cases observed by Bayle were descendants of insane or highly nervous persons, and M. Calmeil believes that *hereditariness* exists in about a third. The influence of *sex* is great, for in the French asylums 1 man in 4 admissions is paralytic, while only 1 woman in 12 is so. The sanguine and plethoric *temperament* is most liable. Of 600 men, the medium *age* was 42, and of 400 women, 41; the disease very rarely occurring before 30. It is most frequently found in those classes of society which indulge in excess of drink, &c. Although we have found that insanity occurs with a third more frequency in summer than in any other season, the result is different as regards paralysis. Thus, of the 921 paralytic patients received at the Bicetre and Salpètrière during four years, nearly an equal number were admitted in the six cold as in the six hot months. Contrary to what occurs in simple insanity, the occasional causes are more often physical than moral. Of these, cerebral congestions, suppression of menses, or hæmorrhoidal discharge, excess of drink or of work, &c. are the most common. Sometimes the disease arises from organic cerebral affections; and, in exceptional cases, from anæmia and exhaustion.

*Symptoms.*—These are exhibited in lesions of motion, sensibility, and the understanding, each being exhibited in three different degrees. Of the *Lesions of Motion* we have (1) *Embarrassments of speech.* These are sometimes scarcely to be observed, being remarkable chiefly by the effort and the suddenness with which the words are articulated. There is a characteristic trembling of the muscles around the mouth, and in a slight degree of the tongue itself. In a second degree there is more hesitation of speech, long intervals separating portions of a sentence, or even of a word, the faulty pronunciation becoming very confused if the patient is excited. In another degree the patient either cannot articulate at all, or does so unintelligibly. (2). *The defective movements of the limbs* usually only manifest themselves after the above. The paralysis also observes different degrees, from a hesitation first in walking, and then a want of precision in employing the hand, both hardly to be detected, until the completest loss of power in all the limbs. (3). *Tremors of the limbs,* which occur in a slight degree in almost all these patients, sometimes prevail to a truly convulsive extent. A remarkable grinding of the teeth is sometimes heard, when no movement of the jaw can be detected. The *Lesions of Sensation,* very slight at first, gradually increase; so that eventually the skin entirely loses its sensibility, and the functions of the special senses become completely destroyed. *Lesions of the Intellect* in some patients consist of simple dementia without delirium, while others manifest the latter, which is, however, always, whatever may be its form, accompanied from the commencement with the marks of dementia. The ambitious form of monomania is the most frequent manifestation of delirium in these cases, but it, as well as the other forms, are maintained, owing to the loss of memory, with much less obstinacy and consistency than ordinary monomaniacs exhibit. The affective faculties become effaced, the patient witnessing the joys or sorrows of his friends with like perfect indifference. The *Nutritive functions* are at first satisfactorily executed, the appetite being, indeed, sometimes voracious. Afterwards this is lost and the patient wastes away. The stools are then past involuntarily; and sometimes, before the sphincters become paralysed, female patients are only observed to befoul themselves at their menstrual periods.

Such is a general picture of the symptoms, and, as regards the order in their advent, I agree with Bayle, that a slight hesitation of speech is the first one, and by observing it I have several times been able to announce the supervention of general paralysis in persons who manifested no other. Next we observe a marked tendency to drowsiness, and then slight dementia, which is first shewn by a weakening of the memory. These persons can now no longer fulfil the duties of their station. They are not competent to even the most simple mental combinations, and incoherence is observed in their discourse. In some, a year or more prior to the accession of the disease, the genital faculties are lost. Among most of those menaced with this paralysis, slight tremors of the limbs, and cerebral congestions, inducing momentary loss of speech, are observed. In almost all cases the paralysis precedes the delirium; but, admitting that occasionally the reverse is the case, must we conclude that *incomplete general paralysis* is always a complication of insanity, which remains the primary and principal disease? We make the following distinction, which we believe an important one. If the lesions of the intellect have only preceded those of motion a short time, and consist only in an ambitious monomania, we must regard the delirium as simply symptomatic of the general paralysis. And we must regard this last as a complication of insanity only in the cases where it follows delirium of several years' duration, and which does not present the special form observed in the paralytic.

As to the *progress* of the disease, it is very irregular. Some patients are detained long at its early periods, and others pass at once to the second stage. Others again, apparently at death's door, recover power of motion surprisingly, only again to relapse. Its *mean duration* seems to be from 18 months to two years, life being more prolonged in females than males. Among the *complications,* epileptic paroxysms are the most frequent, and are often the instrument of the fatal termination. They are much more frequent in men than in women, who seem more liable to a semi-comatose condition. The *prognosis* is of the gravest character. Among the *Anatomical changes* observed, the firm adhesion of the membranes to the more or less softened cortical layer of the brain is the most frequent, M. Calmeil having found it 28 times in 35 cases. In another group of cases, instead of meningeal alterations, collections of fluid are found in the cavities of the brain, while its substance is atrophied and hardened. However, the lesions observed are often very various, and the dependence of the disease upon any special one very unlikely. "Considering that among these alterations none is constant, and that each may be met with in other diseases, we agree with M. Calmeil that this affection cannot be explained by the meningo-encephalitis or the hydrocephalus, but that it is due to some identical modification, the intimate nature of which is unknown, a modification which must exist in all these cases, independently of the lesions described."

The *Diagnosis* is usually not difficult. The cases which may give rise to most embarrassment are, (1) *Delirium Tremens*, accompanied with ambitious ideas and embarrassed speech; but the cessation of the symptoms after a few days' treatment, will remove all doubt. (2) *Diseases of the superior portion of the spinal marrow.* Here the upper limbs are as soon or sooner affected than the lower. There is no embarrassment of speech or mental affection. (3) *Sudden diseases of the brain*, as hæmorrhages, &c. will be distinguished by an ordinary study of the symptoms. There is a class of diseases, which, in our opinion, are identical with general paralysis, and should not be separated from it, viz. the paralysis of the aged, that resulting from the chronic hydrocephalus of adults, or the hydrocephalus following cerebral alterations.

*Treatment* is of no avail, except in the earliest period, and rarely then. If the patient be strong, local, or even small general, bleeding may be advantageously resorted to; employing at the same time purgatives, revulsives, and exutories. In the second period, leeches may be sometimes used, but we must especially rely upon setons behind the neck. By these means we may frequently *delay* the progress of the disease, and can rarely do more.—*Gazette des Hôpitaux*, Nos. 80, 83.

In reference to this interesting disease a short discussion took place not long since at the Society of Practical Medicine of Paris. M. *Brierre de Boismont* observed that, in consequence of its having been stated by some medical men that general paralysis may exist without insanity, he had made researches respecting it in all the large hospitals, and arrived at the following conclusions. 1. General paralysis without mental alienation is excessively rare, since, in examining more than 1500 patients only one distinct instance was discoverable, while such cases constitute about a fourth of the inhabitants of asylums. 2. The paralysis of the dirty patients of the Salpétrière is not identical with this disease. Such patients are all far advanced in age, while general paralysis attacks individuals in the flower of their age, and kills them in a very few years. There is not observed among them, which is an important characteristic so often seen in the insane, those temporary recoveries of power, when even just upon the eve of death. The general paralysis then of the old females of the Salpétrière is only a general enfeeblement of the nervous system, brought on in the progress of age. M. *Colineau* observed that he had seen all the symptoms of general paralysis without insanity, there being simply a weakness of the intellect. In one case, the individual retained his faculties unimpaired to the last. M. *Baillarger* stated that cases of this paralysis are to be met with in the Salpétrière, accompanied by dementia, but without mental alienation, and that he is occupied in preparing a work distinguishing general paralysis from mental alienation. He thinks *progressive paralysis* would be a better denomination for the disease than paralysis of the insane.—*Gazette Medico-Chirurgicale*, No. 24.

### Cancer.

*Opium Dressing.*—M. Tanchou speaks highly of the relief from pain to be obtained from employing the following dressing in cancerous ulcers. Digest, during 24 hours, at a temperature of about 78°, a certain quantity of rough-powdered or bruised opium in a sufficient quantity of water to form a thick paste. Cover the ulcer with this, a line or two in thickness, once or twice a day according to the severity of the pain, and place over it a piece of thin gummed-paper or court-plaister to prevent evaporation.

*Relapse of Cancer.*—True as it is that relapse is much to be feared after operation for cancer, it is no less so that we may sometimes mistake the effects of inflammation for such. M. *Lisfranc* has made the part which inflammation acts in cancer the subject of very attentive study. A woman had her left breast removed recently. Cicatrization at first took place rapidly; when, all of a sudden, the wound broke out again, the surrounding skin assuming a slate-colour, and lancinating pains re-appearing. Here was every appearance of a relapse; but M. Louis ordered a dozen leeches to the margin of the wound, the pains ceased, the slate-colour disappeared, and cicatrization was soon completed.—*Gazette des Hôpitaux.*

---

### Ergotine as a Hæmostatic.

On the occasion of M. *Bonjean's* presenting the *Academie des Sciences* with an account of an additional experiment he has made with *Ergotine*, in which the bleeding from the carotid of a horse, divided through a third of its circumference, was at once arrested by the application of ergotine, M. *Velpeau* delivered the following sensible and pertinent observations:—

"What M. Bonjean says of Ergotine has been said by an infinity of other persons concerning different substances. Hæmostatic means of a *real* efficacy are nevertheless as rare as ever. The error arises from these authors having forgotten two things in their experiments. 1. In animals, the plasticity of the blood is much greater than in man, whence it follows that means which will arrest hæmorrhage in the one, may easily fail to do so in the other. All those who have made experiments on living animals know that, in the horse, the ox, the sheep, for example, the largest wounds of arteries rarely give rise to mortal hæmorrhage. The blood, ceasing to flow almost of its own accord, leads the public and inexperienced authors to believe that it is the *means* or *remedy* employed which has closed the artery. Thus, what powders, waters, liquids, what arcana of every kind, have been vaunted at first as infallible; and then, after a searching examination, rejected as useless! 2. In man, many arterial hæmorrhages also cease either spontaneously or under the exertion of mere compression, without our being obliged to have recourse to the ligature; so that it is easy to attribute to a pretended hæmostatic substance a result which takes place quite independently of its employment.

"I have neither cause or desire to throw any doubt upon the value of M. Bonjean's experiments: but practice has been so often deceived by similar announcements, that it behoves the Academy to accept them with due reserve. I must add, that the practitioners who have tried *ergotine* or the *ergot of rye* have as yet derived nothing conclusive from its use. When, in *uterine hæmorrhage*, the ergot proves useful, it does so by inducing contraction of the uterus, and not by any special action it exerts on the blood or on the arteries. Thus we see the question of surgical hæmostatics is at once a very complex and a delicate one; and we should not receive facts concerning it without a certain degree of distrust, and only give them a very limited publicity, until they have been tested by a more mature examination."—*Comptes Rendus*, 6th July.

---

### M. Ricord on the Treatment of Gonorrhœa.

*The Abortive Treatment.*—As long as there are no acute symptoms, such as pain in urining or during erection, &c. we have only to do with a mere modification of the surface. The passage of the urine only occasions a slight irritation, and as long as the disease has not proceeded farther than this, whatever be its duration, we may still employ the abortive treatment. This is important, for we find practitioners who state that the abortive treatment has not succeeded in their hands, although resorted to within the first twenty-four hours. Time has nothing to do with it; and you will sometimes meet with pneumonias, which in less than twenty-four hours have arrived at their third stage. The *Nitrate of Silver*, used either in substance or solution, is the most powerful modifier of the mucous surfaces we possess. It is not a panacea; but when we have seen the patient early enough, and have been able to ascertain the exact seat of the urethral inflammation, we have frequently applied the substance by means of Lallemand's *porte-caustique* with great success. We give preference however to strong *injections* of this substance, which employed at an early stage, are admirable means. After making the patient pass water and gently squeezing the last drops from the urethra, we must throw in the injection by means of a glass syringe, our object being to make it quickly traverse the entire length of the urethra. If the patient is allowed to slowly inject it, the mucous membrane puckers up, the canal is narrowed, and the fluid does not pass. We should allow it to remain in the urethra about half-a-minute before it flows out. Severe pain in the part is now felt, as if the canal contained pins and needles. We should advertize our patient (only after we have thrown in the injection though) that there will be a temporary increase of pain and discharge, difficulty of urining, together with a more or less abundant exhalation of blood. Such augmentation of the mucoso-purulent discharge may continue for from six to ten hours, the pain however usually ceasing at the end of two or three.

To the severe pains succeeds a complete collapse. The urine is passed with ease, and the discharge sometimes quite dried up. Occasionally the patient is thus cured at once, but unless he observes for a while the greatest precautions, the discharge in a day or two will re-appear. As long as it does not we must rest content; and if it does we employ a second injection, and sometimes are obliged to have recourse to a third one. But, generally, the discharge has become so slight, thin, and mucous, that mere *astringent* injections suffice. However, my experience proves that caustic injections employed, unaided by internal medicines, do not furnish complete results, although I have never seen the ill-consequences described by some as resulting from their employment. Employed alone, then, I reject them: but used in connexion with internal medicines, they constitute an admirable method, and are, without any doubt whatever, the best means we possess for cutting short the progress of this disease.

*Hygienic Treatment.*—If you wish to derive all the advantages possible from the use of these injections simultaneously with the administration of copaiba or cubebs, the hygiene of the patient must be carefully attended to. His diet should be farinaceous, and his drink spare in quantity, so that the urethra may be irritated as little as possible by frequent urining. Tisans must be forbidden if we wish the abortive treatment to succeed; but, when the patient will not be satisfied without them, linseed-tea forms one of the best. Exercise must be taken as seldom as possible, and all thoughts, books, &c. calculated to excite erections, avoided. Intellectual pursuits have at such times a useful effect in driving away lascivious thoughts. A substance I can recommend as an *anaphrodisiac*, acting as powerfully on the genital organs as belladonna does upon the iris, is *Camphor*, and this is the manner in which I employ it. ℞. *Camphor, Thrydace* āā 50 *gr. M. and divide in pil.* 20. Five or six of these are to be taken daily, especially in the evening. For those who cannot take pills, I order 12 grains suspended by yelk of egg, as a *lavement*. Some persons, as I did myself some years since, add opium to the camphor, but this only destroys its sedative effects.

*Internal, indirect, or revulsive medication.*—The most efficacious of medicines are copaiba and cubebs, and after them, but in a far less degree, turpentine and balsams. How do these act? Revulsively sometimes; for the discharge has been evidently arrested after purgation by copaiba or cubebs: but, as Cullerier justly stated, such a cuie is not permanent. The specific, not the purgative action, of copaiba is that which we must seek for. The action of such substances really takes place upon the urinary passages, through which they are eliminated from the system. What best proves that it is by the medicinal urine, loaded with the active principles of these medicines, the disease is cured, is, that it is especially in the cases in which the substance imparts its odour to that fluid that cures take place. I had a patient in whose urethra, affected with discharge throughout its length, was an aperture, dividing it into an outward and posterior portion. Copaiba was given him which cured the discharge in that part of the canal only over which the urine was enabled to flow. Yet we cannot obtain a cure by injecting copaiba or cubebs, but may indeed aggravate the case. There is a change operated by the living chemistry of the body which imparts to the aromatic principle diffused in the urine its curative properties.

*Copaiba* in some patients soon produces nausea and vomiting, and in others purging. In some, again, it induces an erythematous eruption of the skin, usually but of short duration. In a few cases, in which its odour is not perceived in the urine, great disturbance of the nervous centres is observed. *Cubebs* is much more easily tolerated than copaiba, and much more rarely induces ill-consequences similar to those now mentioned. Whichever substance is employed it must be given from the very first in *sufficient doses*, so that the economy may not accustom itself to its presence too easily. For *Copaiba*, the ordinary dose at first, should be 4 drachms per diem, divided into two or three portions. As much as twice or three times this quantity may sometimes be required. Of *Cubebs* the medicinal doses which I prefer, range from 6 to 10 drs. per diem. As long as the dose seems curative we must be content with it, if it do not disagree with the patient, when it should be diminished. It must be continued some time after the discharge has subsided. As to the pharmaceutical forms of these medicines, the less they are modified the more powerful is their action. Cubebs especially should be taken in as nearly its natural state as possible. Few persons, however, can take copaiba in this manner, and the following is the modification of *Chopart's Mixture*, which I have found most beneficial. *Balsam Copaib: Mint Water: Lettuce Water: Orange-flower Water: Syrup of Poppies, of each equal parts. Make into an Emulsion with Tragacanth Powder.* Three spoonfuls are first to be taken daily, and the number increased, as the tolerance becomes established, until 10 or 12 are taken. A little Seltzer water after each dose corrects the tendency of the medicine to rise; but few patients are able to continue it for long. For those who cannot take copaiba by the mouth, it must be given by the rectum; but, so administered, it is a most uncertain remedy, and should never be resorted to, unless no other means present themselves. The following is the formula. *Balsam, 6 or 7 drachms: Decoction of Poppies,* ℥iij. *Yelk of Egg* 1. M. The rectum must be previously emptied by an ordinary enema, and then this one administered almost cold. It should be given at bed-time, and the patient must endeavour to retain it all night; to aid him in doing which, a little opium and camphor may be added to the enema. The introduction of *capsules* is one of the triumphs of pharmacy. I prefer Raquin's gluten capsules to those formed of gelatine, the former containing a little magnesia. I have endeavoured to prepare similar ones less expensively. Thirty parts of Copaiba are solidified with 1½ of magnesia, and then covered with gelatine. With *cubebs* we usually associate a little extract of *rhatany* or frequently powdered *alum* (*i. e.* 2 parts of alum to 30 cubebs). In some lymphatic or chlorotic-looking subjects we combine carbonate of *iron* with the cubebs. The patient's drinks should be in small quantity. Some Seltzer water with a little syrup of oranges, or some infusion of uva-ursi, with a little citrate of iron (6 or 8 parts to 500) and syrup of Tolu, are the best. Cubebs and Copaiba are often given together. I employ them separately: so that if either of these medicines fail me I have recourse to the other: but yet in a few cases where copaiba cannot be supported alone, the mixture of the two is tolerated.

*The Acute Stage.*—Suppose these means have not succeeded in cutting short the disease, and the gonorrhœa is completely established. We must insist upon hygienic precautions, order abundant and diluent drinks, and warm baths (about 92°). These last may be employed for an hour; but for a less time, or not at all, in susceptible patients, in whom they sometimes induce erections. Hip-baths are to be prohibited entirely, as always hurtful. Saline purgatives and enemata, the use of the suspensory, hard cool beds, rigid diet, and the camphor pills, are to be ordered. These means will usually carry the patient through the acute stage, but sometimes powerful antiphlogistics may be required. When leeches are employed, they must be always applied to the perineum, not to the penis. Some practitioners employ caustic injections and copaiba in this acute stage, and they may have met with some cures. In the great majority of cases however they fail, and thus discredit an useful means when judiciously used. I forbid injections of any kind whatever at this period; so, too, in regard to copaiba, &c., it usually does harm or is useless, only disgusting the patient, and preventing his taking it at a more appropriate period. There are, however, certain exceptional cases in which the pain in urining will yield to nothing but copaiba, which then becomes a true sedative. When the antiphlogistics have been discontinued, and the discharge still continues, we may then cautiously have recourse to this substance or the cubebs.

Among the *accidents of the acute stage* we may mention *dysuria*, which, as it is only one stage removed from retention of urine, must be carefully watched. We should employ antiphlogistic means still more energetically, and direct the patient to allow his water to pass while the penis is kept under tepid water. In acute *retention* local or general bleeding at once often affords relief. An enema formed of three ounces of *cold* decoction of poppies and a few drops of laudanum may have the same effect. If these means fail, we must place the patient in a bath at a very low temperature; but here precautions are required; for if the bath does not cause the patient to urine, it augments the retention. If, however, twenty-four hours have elapsed without relief, we must not longer delay the use of the catheter; for the longer we now wait, the more difficult and painful will its introduction prove. Whether the instrument is to be retained or not, will depend upon the duration of the retention and the difficulty of introducing it. Sometimes the retention is produced by the patient restraining himself too long from urining, in dread of the pain this will cause him. The complication of *bubo* and *urethral abscesses* is a simple inflammatory accident, to be met by antiphlogistic measures. They should be opened at the earliest period that suppuration is manifested. *Chordee* is best treated by large doses of camphor, as are also *urethral hæmorrhages*. These last, in a moderate degree, are useful by the local depletion they induce; and when in excess they are best restrained by the external application of ice and slight compression, or by means of cold urethral injections. *Inflammation of the neck of the bladder* is one of the most tormenting affections both for patient and practitioner. The antiphlogistic measures must be vigorously pursued, but they often fail to give relief. A small cold enema, containing a little laudanum, given twice a day, will often relieve the pain in a manner that nothing else can.

*Chronic Stage.*—In the great majority of cases the disease is not cured upon the disappearance of the inflammatory symptoms. As soon as these have lessened we must adopt less relaxing methods. Drinks must be more restricted, and baths left off, as nothing reproduces or prolongs the affection more than an incautious continuance of these last. The camphor must be continued, and copaiba and cubebs resorted to. The use of irritating injections must not be had recourse to, until four or five days after using the balsam; but if the disease then continues stationary, we may have recourse to them. If the inflammatory action, as manifested by pain, has entirely subsided, strong injections of nitrate of silver will suit very well; but this is not usually the case, and the following is that which I then prefer. *Rose Water* 200 *parts, Sulphate of Zinc* 1 *part, Fluid Subacetate of Lead* 1 *part.* Unchemical as it is, it answers exceedingly well. Three times in the 24 hours is quite frequent enough for its use, the patient urining just before employing it. In this way you will cure 98 out of every 100 gonorrhœas. In the few cases which resist we should study the causes of the persistence of the discharge, and apply appropriate means. Sometimes the following vinous injection succeeds in these cases :—*Bordeaux or Roussilon* 150 *parts, Rose Water* 50 *parts, Tannin* 1 *or* 2 *parts.* To this a little *alum* may be sometimes advantageously added. Another excellent injection in quite chronic cases is formed by the *iodide of iron. Distilled Water* 200 *parts, Iodide of Iron* $\frac{1}{10}$*th* or $\frac{1}{5}$*th part.* I do not approve of the *bichloride of mercury*, for, if irritating injections are required, the *nitrate of silver* is the best. The former indurates the surface, and risks the production of stricture. When the discharge resists the strong nitrate injection, it will sometimes yield to a very weak one ($\frac{1}{10}$ p. to 200 parts). In some patients, however weak the injection used, inflammation and increase of discharge result. The patient in such a case should be left alone for a few days, when the discharge will often be found to have ceased.

The treatment of *gleet* or *military gout* is very difficult, as no disease is more obstinate. Its obstinacy generally arises from change of structure, and whenever these patients present themselves, you should *always at once examine the condition of the urethra*, or you may be afterwards accused of producing strictures which already existed when the patient first consulted you. If the canal is free you should try the copaiba and cubebs, and then dried turpentine pills, (from 1 to 2 drachms per diem), giving at the same time decoction of *uva ursi* and syrup of tolu as a drink. Sometimes, astringents and ferruginous preparations are useful; and at other times cauterization of the urethra. Bougies, medicated or otherwise, have often effected a cure. Blistering the perineum or thighs is sometimes of great service. Cold-bathing is another means which may be tried. More frequently than is believed, the persistence of the discharge depends upon prolonged continence; and it is then cured by coition.—*Gazette des Hôpitaux*—No. 82.

---

### M. Gintrac on Diagnosis of Chlorosis.

M. Gintrac's object is to recognize a condition of the economy, characterized principally by paleness of skin, feebleness, and palpitations, but yet quite distinct from that of true chlorosis, and intimately connected with irritation of the digestive canal. The pallor of the skin is of a dead-white instead of the yellowish-green of that disease; and the debility and palpitations are less marked. The

*bruit de souffle* too are less constant. The abdomen is found to be tender on pressure, and the digestive functions are entirely disordered—there being loss of or depravation of appetite, nausea, eructations, constipation or diarrhœa, and sometimes hysterical symptoms. The tongue may be pale, but at other times it is partially red, either at its tip, or in the middle. There is almost always amenorrhœa. M. Gintrac regards these symptoms referrible to the digestive organs as not resulting from pure inflammatory action, but from a complex state of inflammatory irritation and nervous hypersthenia. Preparations of iron and other anti-chlorotic remedies usually aggravate it, while it yields to those of an antiphlogistic and calming character; such as tisanes, milk, infusions of poppies, baths, laxative and emollient enemata, and cupping-glasses to the abdomen. When the symptoms of irritation have ceased, we may resort to tonics and even to iron: but the symptoms then frequently disappear of their own accord.—*Gazette Medicale*, No. 33.

[The distinction here pointed out is an important one: for there can be no doubt that tonics, and especially steel, are frequently hurtful in consequence of sufficient attention not being paid to the removal of a condition of irritation of the digestive organs.—*Rev.*]

